# Prognostic significance of modified lung immune prognostic index in osteosarcoma patients

**DOI:** 10.3389/fgene.2022.972352

**Published:** 2022-10-11

**Authors:** Xuanhong He, Fan Tang, Chang Zou, Longqing Li, Yang Wang, Guy Romeo Kenmegne, Yong Zhou, Minxun Lu, Li Min, Yi Luo, Chongqi Tu

**Affiliations:** Department of Orthopedics, Orthopedic Research Institute, West China Hospital, Sichuan University, Chengdu, China

**Keywords:** osteosarcoma, tumor immune micro-environment, inflammation, prognostic predictive factors, lung immune prognostic index

## Abstract

**Purpose:** Osteosarcoma is the most common primary malignancy of bone with a dismal prognosis for patients with pulmonary metastases. Evaluation of osteosarcoma prognosis would facilitate the prognosis consultation as well as the development of personalized treatment decisions. However, there is limited effective prognostic predictor at present. Lung Immune Prognostic Index (LIPI) is a novel prognostic factor in pulmonary cancers, whereas, the prognostic significance of LIPI in osteosarcoma has not yet been well clarified. In this study, we firstly explore the prognostic role of LIPI and further modify this predictive model in osteosarcoma.

**Patients and methods:** A retrospectively study was conducted at Musculoskeletal Tumor Center of West China Hospital between January 2016 and January 2021. Hematological factors and clinical features of osteosarcoma patients were collected and analyzed. The area under curve (AUC) and optimal cuff-off of each single hematological factor was calculated.

**Results:** In this study, lactate dehydrogenase (LDH), derived neurtrophil to lymphocyte ratio (dNLR), and Hydroxybutyrate dehydrogenase (HBDH) have higher AUC values. LIPI was composed of LDH and dNLR and was further modified by combing the HBDH, forming the osteosarcoma immune prognostic index (OIPI). OIPI divided 223 osteosarcoma patients divided into four groups, none, light, moderate, and severe (*p* < 0.0001). OIPI has a higher AUC value than LIPI and other hematological indexes in t-ROC curve. According to the univariate and multivariate analysis, pathological fracture, metastasis, NLR, platelet–lymphocyte ratio (PLR), and OIPI were associated with the prognosis; and metastasis and OIPI were independent prognostic factors of osteosarcoma patients. An OIPI-based nomogram was also established and could predict the 3-year and 5-year overall survival. In addition, OIPI was also revealed correlated with metastasis and pathological fracture in osteosarcoma.

**Conclusion:** This study first explore the prognostic significance of LIPI in osteosarcoma patients. In addition, we developed a modified LIPI, the OIPI, for osteosarcoma patients. Both the LIPI and OIPI could predict the overall survival of osteosarcoma patients well, while OIPI may be more suitable for osteosarcoma patients. In particular, OIPI may have the ability to identify some high-risk patients from clinically low-risk patients.

## 1 Introduction

Osteosarcoma is the predominant primary malignant bone tumor and mainly affects adolescents and the elderly. The current standard treatment of osteosarcoma includes radical resection and neoadjuvant chemotherapy (Anderson, 2016). With the application of chemotherapy in cancer therapy, the 5-year overall survival (OS) has been improved to 50%–70% (Bielack et al., 2002). However, due to the drug resistance, distant metastasis and/or local recurrence, the outcome of osteosarcoma patients remains dismal (Yan et al., 2016). Therefore, identifying significant factors correlated with prognosis of osteosarcoma patients is urgently needed. Previous studies had reported the prognostic significance of several biomarkers in osteosarcoma and each of them has been correlated with advantages and disadvantages. Traditional prognostic factors including Enneking stage, tumor size, metastasis, and pathological fractures were instructive in making treatment decisions, but they were thought having limited power for prognosis prediction because they just cover single aspect of clinical or pathological features (Yang et al., 2020). New prognostic factors such as the micro-RNAs, long non-coding RNAs, and gene signature were significant in predicting the prognosis and the outcome of osteosarcomas patients. However, the high expenses and inconveniences of those novel factors limited their further clinical application (Liu et al., 2015a; Wang et al., 2015a; Li et al., 2015). Therefore, a simple, accurate, and inexpensive prognostic predictive factor of osteosarcoma patients is urgently required.

Extensive evidences show that cancer-related inflammations play an important role in the progression of malignant tumors (Candido and Hagemann, 2013; Diakos et al., 2014). Targeting of the inflammation pathway has been confirmed as a novel treatment method in prolonging OS (Aggarwal et al., 2009). Due to the diverse roles of inflammation in malignant tumors progression, several biomarkers, including neutrophil–lymphocyte ratio (NLR), platelet–lymphocyte ratio (PLR), lymphocyte-monocyte ratio (LMR), serum lactate dehydrogenase (LDH), and derived neutrophil to lymphocyte ratio (dNLR), were reported valid in predicting the OS and disease-free survival in various cancers (Koh et al., 2015; Pan et al., 2015; Gu et al., 2016; Li et al., 2017).

LDH acts a crucial role in tumor metastasis and proliferation and is associated with the prognosis of osteosarcoma (Augoff et al., 2015; Marais et al., 2015; Yu et al., 2017; Yin et al., 2018; Gong et al., 2019). HBDH is the isoenzyme of LDH and the value of HBDH could reflect the activity of LDH. However, the prognostic effect of HBDH in osteosarcoma patients remains unclear. Defined as absolute neutrophil count/[white blood cell concentration−absolute neutrophil count], dNLR was also a novel inflammation biomarker to measure inflammatory status in cancers (Capone et al., 2018; Mezquita et al., 2021; Yang et al., 2021). According to Mezquita et al. (2018) the combination of baseline LDH and dNLR, also named Lung Immune Prognostic Index (LIPI), is a novel index for predicting the benefit from immune checkpoint inhibitor and predicting OS or progression-free-survival (PFS) in lung cancer (Kazandjian et al., 2019; Sonehara et al., 2020). The role of LIPI was also explored in extra-pulmonary cancers (Auclin et al., 2021; Feng et al., 2021; Obayashi et al., 2022). However, as far as we know, the prognostic predictive ability of LIPI remains unclear in osteosarcoma.

In this retrospectively study, we tend to explore the prognostic significance of the LIPI in osteosarcoma. Additionally, we intend to establish a modified LIPI, the osteosarcoma immune prognostic index (OIPI), for osteosarcoma patients.

## 2 Patients and methods

### 2.1 Patients

From January 2016 to January 2021, all the cases with osteosarcoma in Musculoskeletal Tumor Center of West China Hospital were reviewed. The patients meeting the following criteria were included: 1) patients with high grade osteosarcoma diagnosed by histopathology; 2) patients who presented complete hematological test results before neoadjuvant chemotherapy; 3) patients who received standard treatment at West China Hospital. We excluded: 1) patients who had received neoadjuvant chemotherapy before their first visit in our hospital; 2) patients with hematological diseases; 3) Patients with other malignancies; 4) patients who did not receive standard treatment (patients who are misdiagnosed and inappropriately treated or fail to complete postoperative chemotherapy). Eventually, 223 patients were included in this study and each of them was followed up regularly till death or January 2022. During the follow-up, patients were recommended the outpatient visit every 3 months in the first year postoperatively; every 4 months in the second year; every 5 months in the third year; every 6 months in the fourth and fifth year and yearly thereafter. This study was approved by the ethics committee of West China Hospital and written informed consent was obtained from all participants.

### 2.2 Data collection and analysis

Leukocytes count (Leut#), neutrophils count (Neut#), lymphocytes count (LYMPH#), monocytes count (MONO#), platelet count (PLT), lactate dehydrogenase (LDH), hydroxybutyrate dehydrogenase (HBDH) were extracted from the first blood routine of 223 patients before neoadjuvant chemotherapy. The formulas for calculating NLR, PLR, LMR, dNLR are as follows: NLR = Neut#/LYMPH#, PLR = PLT/LYMPH#, LMR = LYMPH#/MONO#, dNLR = Neut#/(Leut#-Neut#).

In addition, age, gender, tumor site, pathologic fracture status, and tumor metastasis status were collected from the patients’ medical records. OS was calculated from the date of diagnosis to the date of death or last follow-up. In the overall cohort, the optimal cutoff value for each hematological marker was calculated based on the time-dependent receiver operating characteristic (ROC) curve and converted into a binary variable according to the cutoff value.

### 2.3 Establishment and validation of osteosarcoma immune prognostic index

Leukocytes count (Leut#), neutrophils count (Neut#), lymphocytes count (LYMPH#), monocytes count (MONO#), platelet count (PLT), lactate dehydrogenase (LDH), hydroxybutyrate dehydrogenase (HBDH) were extracted from the first blood routine of 223 patients before neoadjuvant chemotherapy. The formulas for calculating NLR, PLR, LMR, dNLR are as follows: NLR = Neut#/LYMPH#, PLR = PLT/LYMPH#, LMR = LYMPH#/MONO#, dNLR = Neut#/(Leut#-Neut#).

In addition, age, gender, tumor site, pathologic fracture status, and tumor metastasis status were collected from the patients’ medical records. OS was calculated from the date of diagnosis to the date of death or last follow-up. In the overall cohort, the optimal cutoff value for each hematological marker was calculated based on the time-dependent receiver operating characteristic (ROC) curve and converted into a binary variable according to the cutoff value.

### 2.4 Construction and evaluation of the nomogram

After the above-mentioned screening process, the prognostic factors were used to construct a nomogram for predicting the OS. For each patient, the total point was equal to the sum of the points of all factors. The link between the total points and the probability of OS were shown at the bottom of the nomogram. The discrimination ability and accuracy of nomograms were evaluated by Harrell’s Concordance Index and calibration curve, respectively. The diagonal acts as a reference line and represents the best prediction. Decision curve analysis (DCA) was used to evaluate the clinical application of the nomogram by estimating the net benefits at different threshold probabilities. The clinical impact curve was also drawn to predict reduction intervention probability per 100 patients. In addition, the constructed nomogram also predicted the overall survival of the validation cohort to assess the stability of the nomogram’s predictive ability.

### 2.5 Exploration of the relationship between osteosarcoma immune prognostic index and clinical characteristics

In all 223 patients, the association between the OIPI and traditional clinical characters, such as tumor site, pathological fracture, tumor metastasis status, was further explored by Spearman correlation analysis.

### 2.6 Statistical analysis

Kolmogorov-Smirnov was used to assess whether continuous variables were normally distributed, and Mann-Whitney *U* test or Spearman correlation analysis was used to assess differences between continuous variables according to the results. Categorical variables were evaluated using the chi-square test and the fisher’s exact test based on the number of individuals in each group. All statistical analyses were conducted using R software, version 4.1.0 (Institute for Statistics and Mathematics, Vienna, Austria). *p*-values < 0.05 were considered to indicate statistical significance.

## 3 Results

### 3.1 Patient characteristics and optimal cut-off values of hematological factors

**FIGURE 1:**
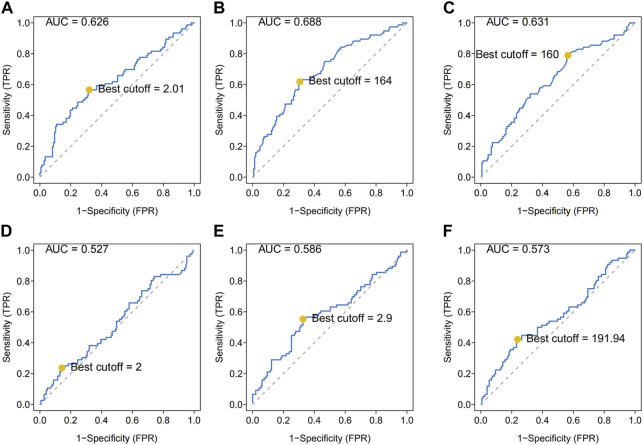
ROC analysis of different hematological biomarkers. **(A–F)** The AUC and best cutoff values of dNLR, LDH, HBDH, LMR, NLR, and PLR were shown, respectively. The vertical axis represents the sensitivity and the horizontal axis represents the 1-specificity. dNLR, derived neurtrophil to lymphocyte ratio; LDH, lactate dehydrogenase; HBDH, Hydroxybutyrate dehydrogenase; LMR, lymphocyte-monocyte ratio; NLR, neutrophil–lymphocyte ratio; PLR, platelet–lymphocyte ratio.

Patient characteristics were shown in Table 1. A total of 223 patients were enrolled in this study including 131 male and 92 female. The age of patients ranged from 7 to 67 years with a mean age of 22 years. Tumors mainly located at the extremities (96.0%) and only 9 tumors (4.0%) located at the extra-extremities. Pathological fracture at diagnosis was found in 25 (11.2%) patients and metastasis at diagnosis was found in 39 (17.5%) patients. The AUCs and optimal cuff-off of LDH, HBDH, PLR, NLR, LMR, and dNLR were calculated. As shown, the AUCs and optimal cuff-off were 0.631 and 160 for LDH, 0.688 and 164 for HBDH, 0.573 and 191.94 for PLR, 0.586 and 2.9 for NLR, 0.527 and 2 for LMR, 0.626 and 2.01 for dNLR, respectively (Figures 1A–F).

**TABLE 1:** Clinicopathological characteristics of patients.

	Patients	OIPI				*p*-value
		None	Light	Moderate	Severe	
Total patients	223	45	72	65	41	—
Age (years)						0.086
>22	67	20	22	15	10	
≤22	156	25	50	50	31	
Gender						0.567
Male	131	29	38	38	26	
Female	92	16	34	27	15	
Tumor location						0.309
Extremities	214	44	70	63	44	
None-extremities	9	1	2	2	4	
Pathological fracture						0.002
Yes	25	5	1	12	7	
No	198	40	71	53	34	
Metastasis						0.003
Yes	39	4	7	14	14	
No	184	41	65	51	27	
LMR						0.001
>2	185	43	62	54	26	
≤2	38	2	10	11	15	
NLR						0.000
>2.9	90	2	25	23	40	
≤2.9	133	43	47	42	1	
PLR						0.006
>191.94	67	8	20	18	21	
≤191.94	156	48	52	47	20	

OIPI, Osteosarcoma Immune Prognostic Index; LMR, lymphocyte-monocyte ratio; NLR, neutrophil–lymphocyte ratio; PLR, platelet–lymphocyte ratio.

### 3.2 Establishment of osteosarcoma immune prognostic index and survival analysis of various hematological factors

**FIGURE 2:**
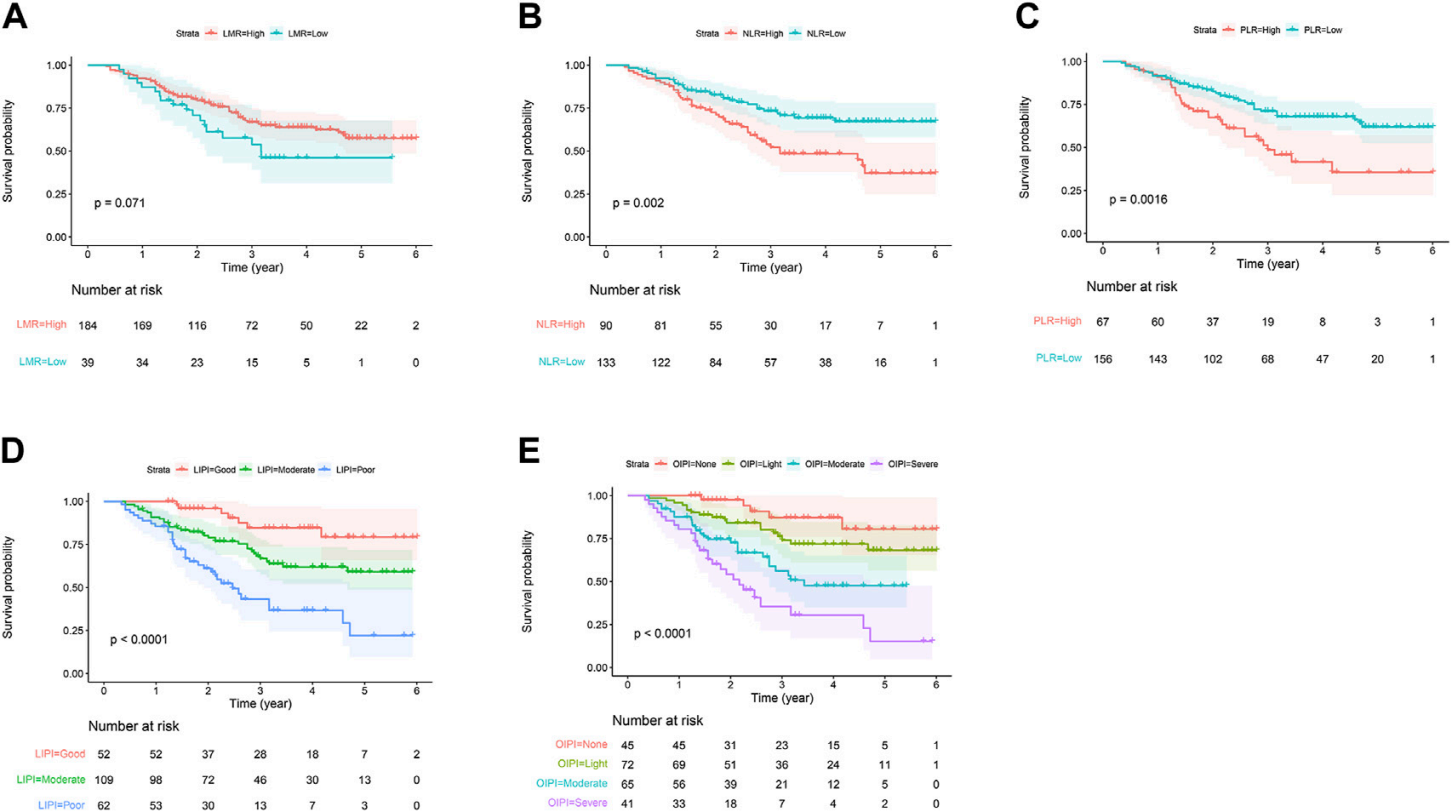
Predictive ability of different hematological biomarkers on OS in 223 patients with osteosarcoma. **(A–E)** Prognostic predictive effect of different inflammatory biomarkers on OS. Cumulative hazard function was plotted by the Kaplan–Meier methodology and the *p* value was calculated with two-sided log-rank tests. According to the logistic regression analysis, the differences between four LIPI or OIPI groups in the survival probability were significant. OS, overall survival; LMR, lymphocyte-monocyte ratio; NLR, neutrophil–lymphocyte ratio; PLR, platelet–lymphocyte ratio; LIPI, Lung Immune Prognostic Index; OIPI, osteosarcoma immune prognostic index.

As shown, several hematologic markers were associated with survival outcomes in osteosarcoma, except for the LMR (Figure 2A). The low NLR group showed a better survival outcome rate than the high NLR score group (*p* = 0.002). The low PLR group showed a better survival outcome rate than the high PLR score group (*p* = 0.0016) (Figures 2B,C).

**FIGURE 3:**
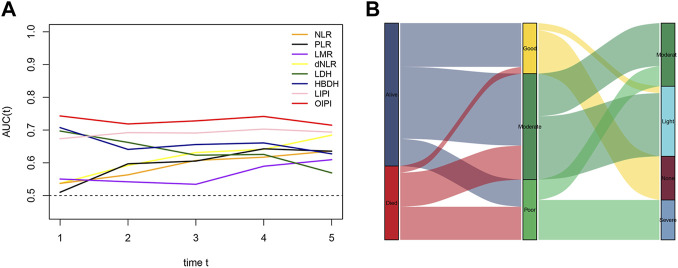
Comparison of different hematological biomarkers in predicting the overall survival. **(A)** The difference of predictive ability was shown in time-dependent ROC curve, in which a larger AUC value meant a better prognostic predictive ability. **(B)** The Sankey showed the difference between LIPI and OIPI in distributing osteosarcoma patients. NLR, neutrophil–lymphocyte ratio; PLR, platelet–lymphocyte ratio; LMR, lymphocyte-monocyte ratio; dNLR, derived neurtrophil to lymphocyte ratio; LDH, lactate dehydrogenase; HBDH, Hydroxybutyrate dehydrogenase; LIPI, Lung Immune Prognostic Index; OIPI, osteosarcoma immune prognostic index.

In the current study, we constructed the LIPI combined with LDH and dNLR referring to previous research (Mezquita et al., 2018). LIPI divided patients into 3 groups: the 1st group of 52 patients who presented good LIPI, 2nd group of 109 patients who presented moderate LIPI, and a 3rd group of 62 patients who presented poor LIPI (Figure 2D). As expected, compared with other hematological, LIPI showed better predictive ability in OS (Figure 3A). However, we found that HBDH was also an effective prognostic factor with AUC value of 164, and performed better in evaluating the OS than other single hematological factors (Figure 3A). Thus, we combined the LIPI with HBDH and developed a new biomarker of osteosarcoma patient, OIPI. OIPI divided 223 osteosarcoma patients into 4 groups: the 1st group of 45 patients who presented none OIPI, the 2nd group of 72 patients who presented light OIPI, the 3rd group of 65 patients who presented moderate OIPI, and a 4th group of 41 patients who presented severe OIPI. OIPI has a good prognostic predictive power that is even stronger than that of LIPI (Figure 3A). To further investigate the distinction between LIPI and OIPI in predicting the OS for osteosarcoma patients, we drew the Sankey with R software. As shown in Figure 3B, patients in good LIPI group were divided into none and light OPI group, while patients in the severe OIPI group were all from patients in the poor LIPI group. As it can be seen, some patients (those who survived) in the poor LIPI group were shunted to the moderate OIPI group rather than the severe OIPI group, indicating that OIPI is more precise than LIPI in identifying osteosarcoma patients with poor prognosis.

### 3.3 Univariate analysis and multivariate analysis

**FIGURE 4:**
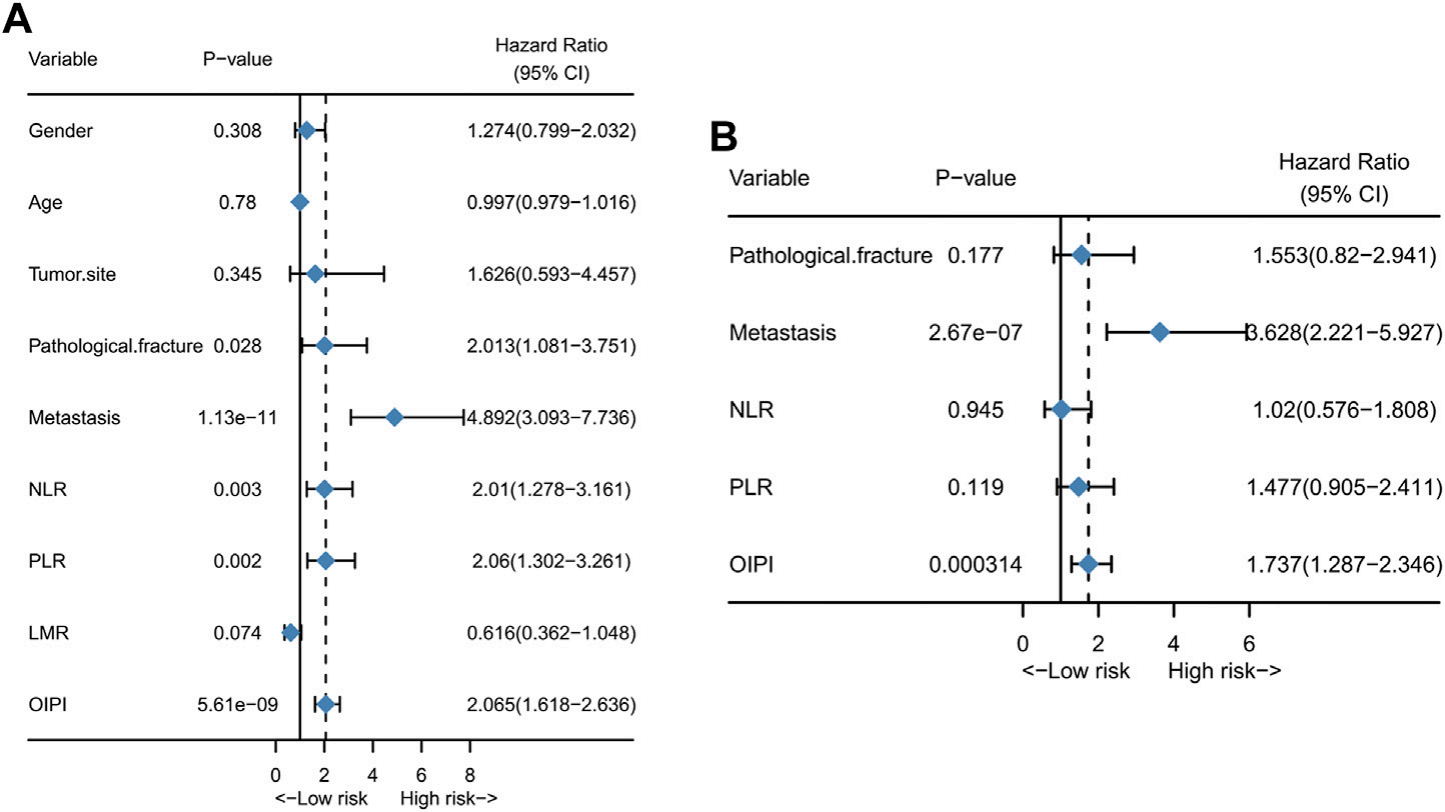
Independent risk factors of OS in 223 osteosarcoma patients. **(A)** Univariate analysis of clinical characters and inflammatory biomarkers. **(B)** Multivariate analysis of significant clinical characters and inflammatory biomarkers in univariate analysis to determinate independent prognostic factors. NLR; neutrophil–lymphocyte ratio; PLR, platelet–lymphocyte ratio; LMR, lymphocyte-monocyte ratio; OIPI, osteosarcoma immune prognostic index.

The univariate analysis exhibited that the pathological fracture (HR = 2.013 (1.081–3.751), *p* = 0.028), metastasis (HR = 4.892 (3.093–7.736), *p* = 1.13e^−11^), NLR (HR = 2.01 (1.278–3.161), *p* = 0.003), PLR (HR = 2.06 (1.302–3.261), *p* = 0.002) and OIPI (HR = 2.065 (1.618–2.636), *p* = 5.61e^−09^) were associated with the OS (Figure 4A). Then the significant values were subjected to multivariate analyses to determine independent prognostic factors. The results showed that both metastasis (HR = 3.628 (2.221–5.927), *p* = 2.67e^−07^) and OIPI (HR = 1.737 (1.287–2.346), *p* = 0.000314) were independent prognostic factors of OS in patients with osteosarcoma (Figure 4B).

### 3.4 Construction and validation of osteosarcoma immune prognostic index-based nomogram

**FIGURE 5:**
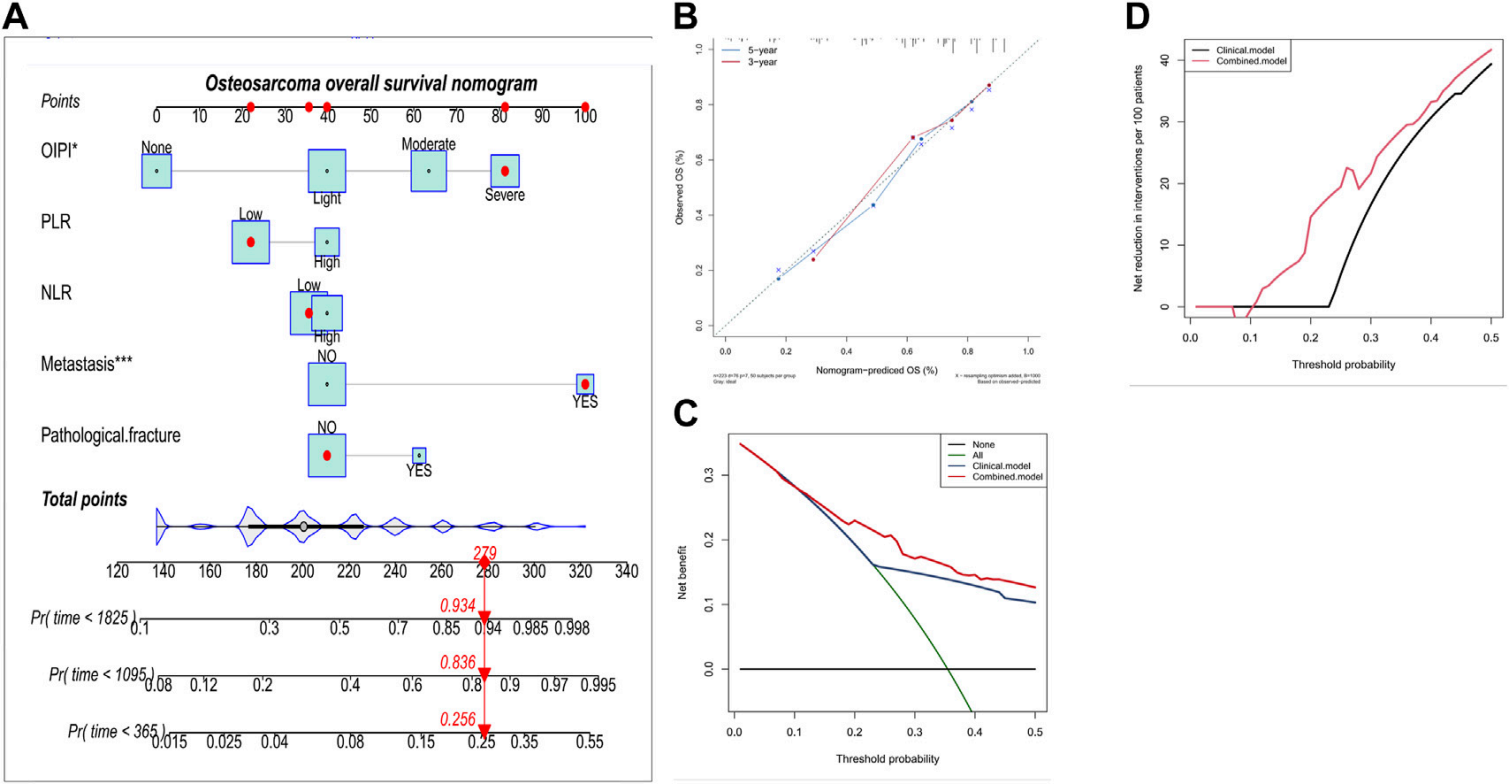
Construction and validation of the osteosarcoma overall survival nomogram. **(A)** The nomogram was constructed by combing OIPI, PLR, NLR, metastasis and pathological fracture and the sum of the scores for each covariate was the nomogram total score. **(B–D)** This nomogram was validated by the calibration curve, decision curve analysis, and clinical impact curve. OIPI, osteosarcoma immune prognostic index; PLR, platelet–lymphocyte ratio; NLR; neutrophil–lymphocyte ratio.

In order to investigate the clinical application of OIPI, we also developed a nomogram combining OIPI with clinical characteristics in patients with osteosarcoma. The hematological indexes (OIPI, PLR, and NLR) and clinical characters (metastasis and pathological fracture) were included in this nomogram to predict the 1-, 3-, and 5-year OS probability for osteosarcoma patients. As shown, cox proportional hazards regression assigned a score based on the hazard ratio for each covariate, and the sum of the scores for each covariate was the nomogram total score (Figure 5A). According to the calibration curve, the 3-year and 5-year OS curve were consistent with the diagonal line in calibration curve, which meant that, this nomogram could accurately predict 3-year and 5-year OS with the C-index of 0.76 (Figure 5B). Moreover, we explored the clinical benefits of the nomogram through DCA and clinical impact curve. The result demonstrated that the combined model (clinical characters and OIPI) could bring significant net benefits over the clinical model (without OIPI) (Figures 5C,D).

### 3.5 The predictive ability of osteosarcoma immune prognostic index compared with clinical characters

**FIGURE 6:**
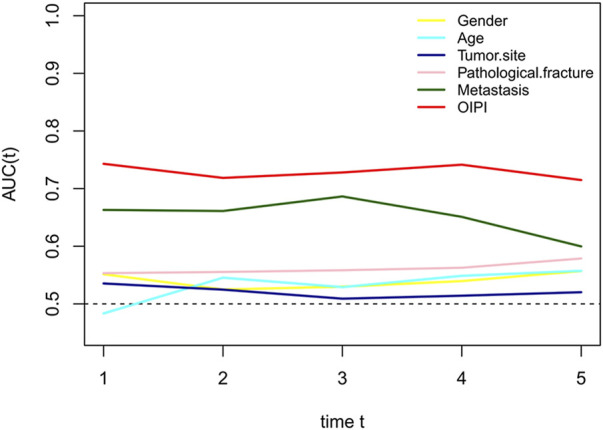
Comparison of the predictive effect between OIPI and clinical characters on OS. A larger AUC in the t-ROC means a better predictive ability. OIPI, osteosarcoma immune prognostic index.

To compare the predictive ability of OIPI with clinical characters including gender, age, tumor site, pathological fracture, and metastasis, we plotted the time-dependent ROC curves. As shown in Figure 6, the predictive effect of the OIPI was significantly higher than that of the clinical characters.

### 3.6 Association between osteosarcoma immune prognostic index and pathological fracture and metastasis

**FIGURE 7:**
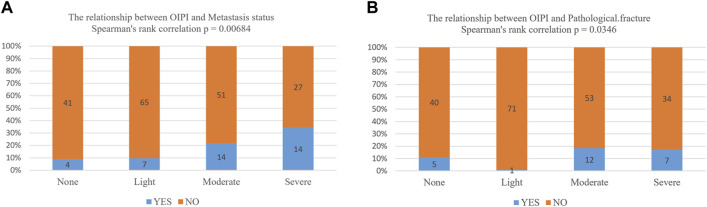
Association between OIPI and clinical characters including metastasis and pathological fracture. **(A,B)** The Spearman’s rank analysis showed that OIPI was related to the metastasis and pathological fracture. OIPI, osteosarcoma immune prognostic index.

Finally, we also explored the relationship between OIPI and clinical characters including pathological fracture and metastasis by Spearman correlation analysis. As demonstrated in Figure 7, OIPI was correlated with metastasis (*p* = 0.00684) and pathological fracture (*p =* 0.0346).

## 4 Discussion

In this study, we developed the OIPI with the combination of LDH, dNLR, and HBDH. OIPI stratify the 223 osteosarcoma patients into four groups: none, light, moderate, and severe. For example, a patient with dNLR>2.01, LDH>160IU/L, and HBDH >164 IU/L was classified as severe OIPI. The OIPI show better prognostic predictive ability over other hematological indexes and clinical features. Besides, our results also revealed that metastasis and OIPI were the independent risk factors of the prognosis in osteosarcoma patients. The significant prognosis risk factors were used to construct a nomogram which could validly predict the 3-year and 5-year OS of osteosarcoma patients. Moreover, OIPI was also closely related to the metastasis and pathological fracture of osteosarcoma patients. Therefore, our findings indicated that OIPI could act as a useful tool to predict the prognosis of patients with osteosarcoma.

Osteosarcoma was the leading cause of tumor-associated mortality in adolescent and children (Ritter and Bielack, 2010). With the advancement of comprehensive treatment, the rate of OS has increased up to 60%–70% in non-metastatic osteosarcoma patients (Bielack et al., 2002). Despite of the advancement of treatment, apparent OS heterogeneity was still observed in osteosarcoma patients. Currently, the traditional clinical features including Enneking staging system, metastasis status, tumor site, histological type, and tumor grade were the main prognosis evaluation factors (Yang et al., 2020). However, those factors have gradually exposed their inaccuracy and inappropriateness during the clinical application, and discrepancy often occurs between those factors and clinical outcomes (Wang et al., 2015b). Recently, several new prognostic factors, including the micro-RNAs, long non-coding RNAs (lnc-RNA), and gene signature were reported effective in the prognosis prediction of osteosarcoma patients (Liu et al., 2015a; Wang et al., 2015a; Li et al., 2015; Li et al., 2021). Most of these biomarkers have a predictive ability, for example, our previous study demonstrated that the metabolic-related gene pairs signature (MRGP) could reliably predict the OS with a high AUC of 0.9 in osteosarcoma patients (Li et al., 2021). Unfortunately, in osteosarcoma, the vast majority of genes have not been validated by independent cohorts and are still away from clinical application. In addition, most of these biomarkers do not have uniform detection methods, such as the expression levels of miRNAs and lnc-RNAs can be affected by the extraction and processing modes (Mathew et al., 2020; Zhong et al., 2020). Indeed, inconsistencies in miRNA and lnc-RNA expression results are frequently reported (Mathew et al., 2020; Zhong et al., 2020). More importantly, the high-cost and inconvenience of detecting these factors limit the further clinical practice.

In contrast, the hematological parameters are derived from blood test results and are low-cost, simple, and convenient to detect. A large number of studies have confirmed the prognostic value of hematological parameters in patients with cancers, such as elevated LDH and ALP implying a poor prognosis in patients with osteosarcoma (Koh et al., 2015; Marais et al., 2015; Pan et al., 2015; Gu et al., 2016; Zumárraga et al., 2016; Li et al., 2017). However, due to the complexity of the tumor microenvironment, a single hematological parameter is not sufficient to fully reflect an individual’s inflammatory status. Nevertheless, there is still a large gap in the predictive ability of these single hematological biomarkers compared with metastasis status. In addition, the predictive stability of these single parameters is not enough and have various clinical significance in different studies, such as the LMR (Liu et al., 2015b; Song et al., 2021) (Figure 2A). As the growing recognition towards inflammatory response and prognosis, it is vital to develop a comprehensive index to evaluate the inflammatory status and to predict the long-term survival rate. Some attempts have been taken to integrate certain significant inflammatory factors in order to evaluate patients’ clinical outcome, such as the establishment of LIPI in lung cancer (Mezquita et al., 2018).

LIPI is a comprehensive inflammatory factor composed of dNLR and LDH (24). LIPI was relevant with inflammatory status and has recently been widely reported as a novel prognostic factor in lung cancer and extra-pulmonary cancer (Mezquita et al., 2018; Kazandjian et al., 2019; Sonehara et al., 2020; Auclin et al., 2021; Feng et al., 2021; Veccia et al., 2021; Xie et al., 2021; Obayashi et al., 2022). More inspiring, studies have shown that LIPI can not only predict the survival but also excellently predict the response to immunotherapy (Mezquita et al., 2018; Auclin et al., 2021; Feng et al., 2021). However, to the best of our knowledge, the prognostic predictive effect of LIPI has never been investigated in osteosarcoma yet. Based on the significant clinical implications for both lung and extra-pulmonary cancers, we hypothesized that, LIPI would also be interesting in predicting the prognosis of patients with osteosarcoma. As expected, our results suggested that LIPI had good predictive ability in predicting the OS of osteosarcoma patients (Figure 3A). The median OS of patients having good LIPI was significantly longer than that of moderate LIPI and poor LIPI, which was consistent with the result reported by Sonehara et al. (2020); Feng et al. (2021). In addition, during the analysis process, we found that HBDH, an LDH isoenzyme, equally showed prognostic significance in osteosarcoma patients (Figures 1B, 3A), and had a good predictive ability with the highest AUC value (0.688) among single hematological parameters (Figure 3A). Given the excellent performance of HBDH in osteosarcoma, we introduced this metric into LIPI and constructed OIPI. We therefore hypothesized that OIPI may be more suitable for patients with osteosarcoma than LIPI.

In this study, OIPI divided 223 patients into four groups, of which 45 patients had none OIPI, 72 patients had light OIPI, 65 patients had moderate OIPI, and 41 patients had severe OIPI (Figure 2E) (*p* < 0.001). Compared with traditional prognostic factors such as metastasis, OIPI divided osteosarcoma patients more evenly; suggesting that OIPI may be able to identify poor prognosis high-risk patients whose metastatic features are not identifiable (poor prognosis in the initial absence of metastasis) (Figure 6). Our findings also elaborated that OIPI performed better than other hematological factors such as LDH, dNLR, NLR, and PLR in predicting OS in osteosarcoma patients (Figure 3A). Most importantly, OIPI does have a higher predictive power than LIPI, as expected (Figure 3A). Compared with LIPI, OIPI is more accurate in identifying patients with poor prognosis. Our results revealed that some of the patients who survived in poor LIPI were redistributed into moderate OIPI group instead of severe OIPI group, while all patients who died in poor LIPI were distributed into severe OIPI group (Figure 3B). This led to the hypothesis that, OIPI is more likely to identify osteosarcoma patients who have a real poor prognosis. Moreover, the combination of dNLR, LDH, and HBDH can further reduce the potential bias, as each individual indicator may be affected by various factors. Our results suggested that OIPI is indeed more suitable for osteosarcoma patients than LIPI. In the other hand, OIPI has the advantage of being low cost and is as easily accessible as other hematological factors. Therefore, we believe that OIPI may be more suitable for clinical application than other hematological factors.

Inflammation related to cancers has been recognized as the seventh landmark of cancers (Mantovani et al., 2008). Inflammation predisposes to tumor development and promotes various stages of tumor initiation, growth, progression and metastasis (Greten and Grivennikov, 2019). Through engaging the dynamic and extensive interactions with cancer cells and surrounding stromal, inflammatory cells participate in the formation of the inflammatory tumor microenvironment (Greten and Grivennikov, 2019). The dual role of neutrophils in inhibiting or promoting cancer cell growth and metastatic spread remains controversial. But in general, neutrophils are associated with the metastasis at nodal site, tumor grade, and tumor stage for its high intra-tumoral density in solid tumors (Masucci et al., 2019). In contrast, lymphocytes in solid tumors are thought to participate in antitumor immunotherapy by secreting cytokines and inducing apoptosis of tumor cells, and there have been lots of studies evaluating their predictive value in different immunotherapies and chemotherapies (Teixidó et al., 2015; Ingold Heppner et al., 2016; Tas and Erturk, 2017). Platelets protect circulating tumor cells from lethal attack by the immune system or other proapoptotic stimuli, and provide signals to establish a pro-metastasis niche environment, ultimately promoting tumor growth and metastasis (Haemmerle et al., 2018). As a classical prognostic factor, LDH could reflect systemic cancer burden and predict the outcomes of numerous cancers, in which an elevated LDH was correlated with the poor prognosis of osteosarcoma patients (Walenta and Mueller-Klieser, 2004). dNLR is a more responsive indicator of systemic inflammatory status than NLR as dNLR includes monocytes and other granulocytes. The predictive potential of dNLR has been demonstrated in a variety of cancers (Capone et al., 2018; Mezquita et al., 2021; Yang et al., 2021). In non-colorectal gastrointestinal cancer, Li et al. (2020) reported that higher level of dNLR was associated with reduced OS in patients with non-colorectal gastrointestinal cancer. To our knowledge, this study is the first to explore this biomarker in osteosarcoma. Our results suggested that elevated dNLR (>2.01) was also correlated with the poor outcome of osteosarcoma patients (Figures 1A, 2A). As the basic components of OIPI, the elevated LDH, dNLR, and HBDH are associated with the poor outcomes in osteosarcoma.

It must be acknowledged that our study has some limitations. First, this was a single-center study, which was retrospective and may have caused selection bias. Second, this study did not fully explore the predictive potential of OIPI. To our knowledge, two studies with large sample sizes have affirmed the prognostic value of LIPI in predicting response to immunotherapy in non-small cell lung cancer. Therefore, it is reasonable to assume that OIPI may be able to predict the response to immunotherapy in osteosarcoma. However, as the first to explore the prognostic ability of LIPI and OIPI in osteosarcoma, this current study lays a foundation for evaluating LIPI and OIPI in predicting the response to immunotherapy in osteosarcoma. Finally, the prognostic value of HBDH in osteosarcoma still needs further validations. This study preliminarily explored the prognostic value of HBDH, an isoenzyme of LDH, a classical marker for predicting the prognosis of cancer patients. Surprisingly, HBDH performed better than LDH in our cohort. However, studies on the prognostic value of HBDH in cancer patients are very scarce. In osteosarcoma, only our study has reported the prognostic value of HBDH. Further studies are therefore needed to clarify the predictive power of HBDH in patients with osteosarcoma or even cancer.

## 5 Conclusion

In conclusion, this present study is the first to construct an OIPI that may be more suitable for osteosarcoma patients based on LIPI and practical hematological markers in osteosarcoma. Our results revealed that both LIPI and OIPI could predict the overall survival of osteosarcoma patients well, and OIPI had a better predictive ability than other hematological parameters. In particular, OIPI may have the ability to identify some high-risk patients from clinically low-risk patients. Further studies are needed to validate our conclusions, especially the value of LIPI versus OIPI in predicting response to immunotherapy in osteosarcoma patients.

## Data Availability

The original contributions presented in the study are included in the article/Supplementary Material; further inquiries can be directed to the corresponding authors.
